# Multiple-agent promotion in a grocery store: effects of modality and variability of agents on customer memory

**DOI:** 10.3389/frobt.2024.1397230

**Published:** 2024-12-05

**Authors:** Takato Mizuho, Yuki Okafuji, Jun Baba, Takuji Narumi

**Affiliations:** ^1^ AI Lab, CyberAgent, Tokyo, Japan; ^2^ Graduate School of Information Science and Technology, The University of Tokyo, Tokyo, Japan; ^3^ Graduate School of Engineering Science, Osaka University, Osaka, Japan

**Keywords:** social robot, virtual agent, multiple-context effect, social presence, memory, field study

## Abstract

The use of social robots for product advertising is becoming prevalent. Previous studies have demonstrated that social robots can positively impact *ad hoc* sales recommendations. However, the essential question of “how effectively customers remember the advertised content” remains unexplored. To address this gap, we conducted a field study where physical robots or virtual agents were stationed at two locations within a grocery store for product promotion. Based on prior research, we hypothesized that customers would exhibit better recall of promotional content when it is heard from different agents rather than the same agent. Moreover, we posited that customers would exhibit more favorable social attitudes toward physical robots than virtual agents, resulting in enhanced recall. The results did not support our hypotheses, as no significant differences were observed between the conditions. However, when the physical robot was used, we observed a significant positive correlation between subjective ratings such as social presence and recall performance. This trend was not evident when the virtual agent was used. This study is a stepping stone for future research evaluating agent-based product promotion in terms of customer memory.

## 1 Introduction

Service robots are becoming more prevalent in commercial settings, where they are expected to deliver information and promote sales (Niemelä et al., 2017). Previous studies have suggested that robots are effective in *ad hoc* persuasion and recommendation (Song et al., 2023). However, research must be conducted to address another critical facet of advertising effectiveness from a long-term perspective, that is, how well customers remember the advertised content. Moreover, methodologies must be established to enhance this recall capability.

This study focused on the multiple-context effect (Smith and Vela, 2001; Mizuho et al., 2023a) to enhance the memory retention of content that is promoted using agents. This effect posits that recall performance improves when information is presented in various contexts. Previous research using agents indicated that remote lectures using multiple instructor agents could improve the memory of students on lecture content when compared to the memory measured after using a constant agent (Mizuho et al., 2023a). Drawing inspiration from this line of research, we hypothesized that when different products are promoted at two locations in a grocery store, customers would demonstrate better recall when these recommendations were delivered by different agents rather than when the same agent advertises both products.

Notably, most field studies employing service agents have primarily used robots (Song et al., 2023; Okafuji et al., 2023). However, we must consider the inherent drawbacks of robots, particularly with respect to hardware and maintenance costs. In contrast, employing virtual agents that are displayed on a monitor offers a practical and cost-effective solution. Consequently, several laboratory experiments have compared the effects of physical and virtual agents (Li, 2015). Similarly, the effects of serving customers with a combination of physical and virtual agents on service employees with disabilities have been studied in the field (Hatada et al., 2024). However, to the best of our knowledge, while studies have explored the effects of virtual agents in online shopping (Tan and Liew, 2020), no prior field studies in the real-world store involving virtual agents have been conducted in the domain of product promotion. Therefore, this study addresses this gap by exploring whether there are differences in subjective evaluations and memory performance when using a physical agent versus a virtual agent.

The contributions of this study are as follows. (1) We proposed a method using multiple agents to enhance the memory retention of advertising content, which is an immature topic in the field of advertising using service agents. Although the research did not reveal any differences in memory performance, this study is an initial step toward advancing future research on the effect of agent-based promotion on customer memory. (2) When comparing the outcomes of using a robot versus a virtual agent, we observed no significant differences in memory performance or subjective ratings. While the effects of agent modality on social attitudes have been consistently observed in laboratory experiments, to the best of our knowledge, our study is the first to validate these effects in the field, suggesting that the effects may not be as pronounced in a stimulus-rich field. (3) A significant positive correlation was exhibited between the social presence of agents and memory performance when physical robots were used. This trend was not evident in the virtual agent condition. To the best of our knowledge, this study represents a pioneering field study involving product promotions by virtual agents in the real-world store. Therefore, this finding will provide a clue for future research that examines the differences among agent modalities.

## 2 Related work

### 2.1 Service agent in a field

Several attempts have been made to install service robots in actual commercial settings to facilitate sales. Notably, field studies have been conducted in various venues, including shopping malls (Shiomi et al., 2013; Song et al., 2021), department stores (Watanabe et al., 2015), and bakeries (Song et al., 2023; Okafuji et al., 2023). The introduction of robots into real environments has been favorably received by customers and store clerks, who expect them to be responsible for promoting products and delivering information (Niemelä et al., 2017). Additionally, earlier studies have employed virtual agents in the context of online shopping or e-commerce, revealing that an agent’s design (e.g., gender, attire, or expertise) can impact user behavior (Tan and Liew, 2020; Lunardo et al., 2016).

These prior studies have focused on assessing the *ad hoc* effects of sales promotions. However, research on another important aspect from a long-term perspective, that is, how effectively customers remember promotional content, is still required. Although Iwamoto et al. (2021) reported certain findings related to the memory of product information, this was not the primary objective of their study. To our knowledge, no prior studies employing agents in field settings have thoroughly investigated how customers retain advertising content and developed strategies that can enhance this memory retention. Consequently, further research must be urgently conducted to fully harness the potential of utilizing agents in product promotion.

Furthermore, the use of multiple robots has also been considered (Kamei et al., 2010; Song et al., 2023; Barbareschi et al., 2023). For example, Song et al. (2023) introduced an innovative approach wherein one agent operated outside and another within a bakery to share the roles. Notably, some studies used the same robots (Song et al., 2023; Tae et al., 2021; Barbareschi et al., 2023), whereas others used robots that varied in terms of clothing and voice (Kamei et al., 2010; Salomons et al., 2021). However, no studies have directly compared the effectiveness of employing identical robots versus different ones for promotional purposes.

In this study, we conducted a field study within a grocery store by placing service agents at two locations to promote products and then assessing the customer memory related to the advertised content. Focusing on the multiple-context effect described in Section 2.2, we manipulated whether the two agents were identical or distinct. Moreover, while many field studies have used physical robots, this study explored the difference in advertising effectiveness between physical and virtual agents, as outlined in Section 2.3.

### 2.2 Multiple-context effect

This study focused on the multiple-context effect for enhancing the memory of advertising content. The multiple-context effect is a phenomenon in which recall performance improves when information is presented in diverse situations (Smith and Vela, 2001). The underlying mechanism can be explained as follows. Human memory automatically incorporates incidental environmental information, such as places, sounds, and smells, which serves as a cue for later retrieval (Smith and Vela, 2001; Tulving and Thomson, 1973). When learning occurs within multiple environmental contexts, more cue stimuli are encoded within memory, thereby increasing the accessibility to the memory. This explanation is referred to as the encoding variability hypothesis (Bower, 1972).

It should be noted that two patterns of the phenomenon are both called multiple-context effects. One is an effect when learning the same information repeatedly in various contexts (Type 1) (Smith et al., 1978; Smith and Handy, 2014). The other type is an effect when learning different pieces of information in different contexts (Type 2) (Smith, 1982; Mizuho et al., 2023b). While it is unclear that they can be treated as the same phenomenon, both can be explained using the encoding variability hypothesis or its modification. Regarding Type 1, a single piece of information is associated with various contextual cues. Thus, there are many routes for retrieval, and as a result, the information can still be accessed even if one of the routes becomes unusable due to forgetting. Regarding Type 2, various contextual cues are encoded in association with different information. Then, there are many routes for retrieval. Therefore, even if one of the routes becomes unusable due to forgetting, a large part of the whole information will be accessible. Type 1 is more well-known than Type 2. However, the target of this study is Type 2 because in the grocery store field, Type 2, in which different products are advertised in different places, is more suitable than Type 1, in which the same product is advertised in two places. This paper focuses on the Type 2 version of multiple-context effects and will not cover Type 1.

Early studies into the multiple-context effect manipulated the environmental context of the room in which the word list was learned; Smith (1982) showed that in a 100-word memorization task, a recall was better in a two-room condition, in which participants learned 50 words in each of two distinct rooms, than in a one-room condition, in which they learned 100 words in a single room. Furthermore, the recall was best under the four-room condition, where the participants learned 25 words in each of four rooms. Smith and his colleague corroborated this effect in similar experiments (Smith, 1984; Smith and Rothkopf, 1984).

Recently, researchers have identified that the multiple-context effects extend beyond the traditional room context. For example, Mizuho et al. (2023a) conducted a study examining the impact of virtual avatar changes during a 90-min remote lecture. Their findings revealed a higher proportion of correct responses in a recall test when the lecturer switched between four different avatars rather than delivering the entire lecture using a single avatar. Notably, in their study, only the appearance of the lecturer avatar was manipulated, while the voice remained consistent with that of the original lecturer. Moreover, Tapia et al. (2022) reported a multiple-context effect using a reading voice when learning new vocabulary. Their results suggest that even variations in just the voice can induce a multiple-context effect, further highlighting the adaptability of this phenomenon. The agent’s appearance and voice provide contextual information and demonstrate a multiple-context effect, as the agent is not merely an artifact but establishes a social relationship with the user (Reeves and Nass, 1996). Changing the agent can alter the social context of “from whom the information was received” and may yield an effect similar to that of manipulating a place-based context.

In this study, we pursued a multiple-context effect by varying the agents placed within a real store. Specifically, we varied the appearance and voice of the agents based on the previous studies (Mizuho et al., 2023a; Tapia et al., 2022). To further increase the agent divergence, we changed the speed and size of their movements, taking inspiration from previous studies on personality traits of the robot (Lee et al., 2006b). It should be noted that the multiple-context effect can be obtained by manipulating the primary context that defines the episode, regardless of whether other minor contexts are constant or changing. For instance, in a previous study by Mizuho et al. (2023a), the room the students were in and the monitors they were using were constant. However, the multiple-context effect occurred when the lecturer avatar, which was the main element of the remote lecture experience, was changed. Similarly, in this study, while the global context of shopping in a supermarket did not change, and the local location changed while walking, we posited that the multiple-context effects could be obtained by manipulating the agent’s appearance, a key element of the experience. While Mizuho et al. employed virtual agents, our research would expand upon this finding by comparing the use of virtual agents with physical robots.

### 2.3 Physical robot versus virtual agent

Two prevalent methods for implementing social agents involve physical robots and virtual agents. Extensive research has demonstrated that both modalities effectively influence social behaviors and attitudes of users during interactions (Li, 2015). However, practical differences exist in the implementation of them. Employing physical robots involves hardware and maintenance costs, whereas virtual agents are more cost-effective because they can be achieved using just a monitor. However, robots possess a unique attribute, their physical presence, which has the potential to impact user cognition more than virtual agents (Li, 2015). Therefore, the differences between the effects observed when employing a robot agent and those achieved when using a virtual agent must be discerned and employed appropriately based on the purpose and situation.

A comprehensive review indicated that physical agents elicit more favorable behaviors and attitudes from users than virtual agents (Li, 2015). For example, studies have shown that, when compared to virtual agents, physical robot agents demonstrate a positive impact on the susceptibility of users to persuasion (Bainbridge et al., 2011; Kiesler et al., 2008), enhance the enjoyment experienced during interactions (Kose-Bagci et al., 2009; Wainer et al., 2006; Wainer et al., 2007), attraction (Kiesler et al., 2008; Kose-Bagci et al., 2009; Wainer et al., 2007), perceptions of intelligence (Kose-Bagci et al., 2009), trustworthiness (Kiesler et al., 2008), utility (Wainer et al., 2007), and the sense of social presence (Fasola and Matarić, 2013; Lee et al., 2006a). Whether a physical or virtual agent is more suitable will vary depending on the task and situation (Shinozawa et al., 2005). However, overall it appears that physical agents tend to produce more positive effects (Li, 2015). Furthermore, Lee et al. (2006a); Lee et al. (2006b) suggested that social presence plays a mediating role in shaping the overall impression of agents, their attractiveness, and perceived intelligence. This underscores the critical significance of social presence in agent-related research, highlighting its role as a key determinant of user experience and perception.

In this study, we compared the effects of physical and virtual agents in a field. As all of the previous studies reviewed above were conducted in laboratory settings, it is unclear whether similar results would be obtained in a field with many stimuli and noise, i.e., many environmental cues obtained from sources other than the agents. Then, to analyze the differences in impressions given by virtual and robot agents in a complex context and the resulting effects on memory, we measured the social attitudes toward the agents, including social presence, and explored the relationship with memory retention. To our knowledge, this is the first study that placed virtual agents for product promotion in a real-world field and compared the effectiveness with that of physical robots.

## 3 Materials and methods

### 3.1 Field and product promotions

**FIGURE 1:**
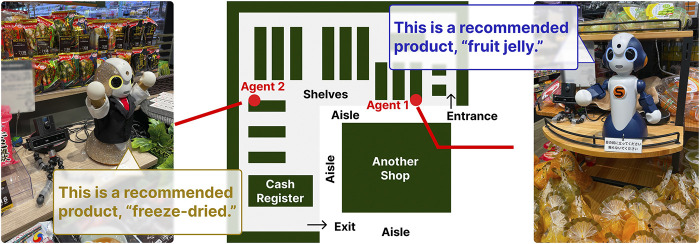
We placed agents at two locations in a grocery store to promote different products. We hypothesized that placing different agents would lead to better recall than placing the same agents. We also investigated the effect of agent modality, that is, the use of physical or virtual agents, on customer memory.

Considering ecological validation, the field study was conducted during the business hours of a grocery store in Osaka, Japan. The store was located on the basement floor, near a busy train station. Figure 1 shows an abstract map of the store. The store had an L-shaped configuration, with customers entering at one end and exiting at the other. One agent was positioned near the entrance (Agent 1), while the other was placed toward the middle of the store (Agent 2). This particular layout was determined in consultation with the store owner and chosen from various placement options. Notably, the two agents promoted different products: fruit jelly at the first position and freeze-dried products at the second position. These products were selected in response to a request from the store owner from among the items actually sold in the store.

The promotional content included details such as the product name, name of the manufacturer, taste, and recommended situations. Each promotion consisted of eight sentences that the participants had to remember. The selection of this number, eight, was determined based on two key considerations. First, we assumed that the maximum duration a customer could pause without discomfort was approximately 1 min. Second, we fine-tuned this number through preliminary tests with our lab members to ensure an appropriate difficulty. Consequently, the promotional audio for each product lasted approximately 40 s. Notably, the same promotional sentences were used across all variations of agents.

### 3.2 Design

Participants in the field study listened to promotional presentations from agents at the two locations while shopping at a grocery store (Figure 1). We evaluated their ability to recall these promotions after leaving the store. Our field study employed a 2×2 between-participants design, denoting two primary factors. The first factor was the modality of the agents, which included two levels: physical and virtual. The second factor was the context factor, which comprised two levels of whether the agents placed at the two locations were the same or different. In the same context condition, we divided participants into two subgroups and systematically counterbalanced the choice of the robot (Section 3.5). Half the participants in this condition encountered a navy-colored agent at both locations. In contrast, the remaining half experienced a gold-colored agent at both locations. Similarly, we organized participants into two subgroups in the different context conditions to ensure a counterbalance. One group encountered a navy-colored robot at the first location, followed by a gold-colored robot at the second location; conversely, the other group experienced vice-versa settings. Consequently, four sub-groups were established for the context factor, represented as combinations of the first and second agents: navy-navy, gold-gold, navy-gold, and gold-navy.

### 3.3 Measurements and hypotheses

Our primary measurement was the proportion of correctly recalled information in the test conducted immediately after leaving the store. The responses were scored based on the inclusion of predetermined information, which was established by the first and second authors in consultation. Specifically, we overlooked minor mishearing and gave points if the participant mentioned the topics the agent was talking about (e.g., product name, manufacturer name, flavor, or recommended situations). For each product, the proportion of items that participants could recall out of a total of eight items was calculated as the recall rate. Based on the multiple-context effect, we hypothesized that promotions using different agents would yield a better recall than those using the same agent. Moreover, we hypothesized that the physical agents would enhance the recall when compared to that of the virtual agents owing to their high social presence. As a further exploratory hypothesis, we posited that physical agents would exhibit a greater multiple-context effect than virtual agents. Thus, our hypotheses were as follows.

**H1:** The different context condition would achieve better recall than the same context condition.
**H2:** The physical agent condition would achieve better recall than the virtual agent condition.
**H3:** The differences in recall between the physical-same and physical-different conditions would be larger than those between the virtual-same and virtual-different conditions.

Moreover, we employed questionnaires to collect subjective evaluations regarding the agents. To measure social presence, we used a questionnaire consisting of five questions that were adopted by Li et al. (2016), which were originally derived from a study conducted by Lee et al. (2006b). Participants responded on a scale ranging from 1 to 10 for each question. Except for one reversed item, 1 represented the lowest presence, and 10 represented the highest presence. After reversing the rating of the reversed item (e.g., converting 2 to 9), we computed the average of the five responses to derive a score representing the social presence. We hypothesized that physical agents would exhibit higher social presence than virtual agents (Fasola and Matarić, 2013; Lee et al., 2006b). We also posited in an exploratory manner that a higher level of social presence would correspond to an increased recall. These hypotheses are presented below.

**H4:** Social presence would be higher in the physical agent condition than in the virtual agent condition.
**H5:**A higher social presence would correspond to a better recall.

Furthermore, to understand the interaction of the participants with the agent, we adopted an approach outlined by Li et al. (2016) to assess the attraction and overall impression that the participants experienced regarding the agents. Participants provided ratings on a 10-point scale, ranging from 1 to 10. The measurement of attraction comprised four items, and we computed the average of these responses to determine the attraction score. Moreover, following the methodology employed by Okafuji et al. (2023), we assessed the perceptions of participants regarding the intelligence, usefulness, impact, and trustworthiness of the agents. This assessment was conducted using a seven-point scale ranging from 1 to 7. We added a question to assess how well the participants concentrated on the agents using a seven-point Likert scale. Similar to the measurements of social presence, we explored the potential relationship between these indicators and recall performance.

Finally, participants evaluated the degree of similarity between their experience in this study and their usual shopping experience on a 7-point scale ranging from 1 to 7. The assessment was used to identify a potential limitation, specifically, how much the unique instructions explained below for conducting the memory experiment (e.g., walking slowly) prevented us from measuring the natural cognition of participants.

### 3.4 Participants

We employed 56 participants, consisting of 28 males and 28 females, with an average age of 35.5 (*SD* = 11.9) years. We recruited them using a staff recruitment agency. The sample size was determined using G*Power (Faul et al., 2007), which yielded a minimum of 52 participants to be required for a large effect. Accordingly, as we considered eight subgroups in this field study, the sample size had to be a multiple of eight. Therefore, the final sample size was determined to be 56.

The participants were randomly assigned to the four conditions to make each group as equivalent as possible, considering the age and gender balance. Consequently, the physical-same condition included seven males and seven females with an average age of 38.7 (*SD* = 13.3) years, whereas the physical-different condition included seven males and seven females with an average age of 35.1 (*SD* = 13.3) years. Moreover, the virtual-same condition included seven males and seven females with an average age of 36.6 (*SD* = 12.0) years, whereas the virtual-different condition included seven males and seven females with an average age of 31.6 (*SD* = 8.5) years. The participants received approximately $20 after completing the field study. The experimental protocol was approved by the Research Ethics Committee of Osaka University (Reference number: R1-5-9).

### 3.5 Physical and virtual agents

**FIGURE 2:**
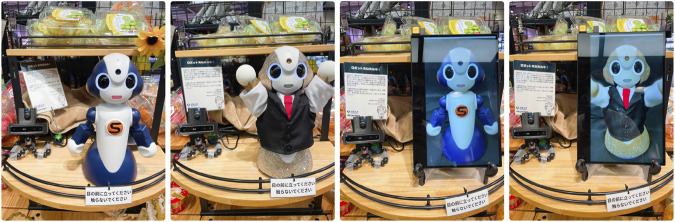
Physical and virtual agents used in the field study. The leftmost robot was navy in color and had a male voice and slow and small gestures. The next robot was decorated with gold-colored rhinestones wearing a black suit-like outfit and had a female voice and fast and large gestures. The virtual agents were implemented by projecting a video of them onto a monitor, making the agents appear about the same size.

The social robots used in the field study were Vstone Sota^1^, as shown in Figure 2. Sota is 280 mm tall and 140 mm wide. It has two movable joints in each arm, and the head and torso can rotate, allowing it to express various gestures. Moreover, LED lights are built into the eyes and mouth, allowing minimal facial expressions such as eye color to express emotion and mouth lighting in sync with the voice. Sota was connected wirelessly to a nearby Windows-based PC, and a Python program on the PC controlled its behavior. This program used depth information obtained from an Intel RealSense D415 camera and detected a human stopping in front of the robot by MoveNet. Upon such an occurrence, the robot initiated the promotional speech and behavior. The audio was output from an Emeet M2 speaker located behind the robot.

To manipulate the context factor of whether the agents at the two locations were the same or different, we prepared two distinct Sota robots: a navy-colored robot and gold-colored robot decorated with rhinestones (Figure 2). They were designed based on the methods described in previous studies to create a robot that is perceived as having different personality traits (Lee et al., 2006b). Therefore, the robots were different in terms of body color (navy versus glittering gold), attire (none versus black suit), eye color (blue versus yellow), voice (male versus female), and gestures (small and slow versus large and fast). We created the voices using a vocal synthesizer, VOICEVOX^2^.

The virtual agents were implemented by projecting a video of Sota onto a display, as illustrated in Figure 2. While we initially considered utilizing three-dimensional computer graphics (3DCG), this option was unavailable due to the absence of an official 3DCG model for Sota. Notably, several prior studies have indicated the lack of a significant difference in cognitive impact between using a video of an actual robot and a 3DCG representation (Li, 2015). The video footage was recorded using a smartphone with a resolution of 1080×1920 pixels and subsequently projected onto a display with the same resolution. We adjusted the display size to be similar to the size of the physical robot. This display was connected to a PC, with video control being managed by a Unity application (ver. 2019.4), which communicated via UDP with the same human detection program used in the physical agent condition. The equipment setup, including the Realsense and speaker, remained consistent with that used for the physical agent.

### 3.6 Procedures

The study was conducted between 10 a.m. and 7 p.m. during the grocery store’s business hours. Therefore, many other customers were shopping during the field study in addition to the participants. The time of the study was determined in consultation with the store owner to avoid busy times, such as opening hours (9:30 a.m.–10:00 a.m.) and closing hours (-10 p.m.). Therefore, the store was not so crowded during the field study, and there was no difficulty in listening to the agents’ talk or staying in front of the agents.

Participants were gathered in groups, ranging from two to six individuals, in an open space outside the store. They were briefed on the details of the experimental procedure. Importantly, during this initial briefing, they were not informed about the memory test. Instead, they were instructed to engage in shopping and were handed a store map indicating the two specific locations where they were required to listen to the advertisements. Such instructions had the potential to reduce the ecological validity of the verification but were necessary because it was impossible to compare memory performance without guaranteeing that the participants would listen to the agent’s advertisements at the two locations. Other instructions were given maximum consideration to simulate a situation similar to the usual shopping experience. For instance, the fact that the agents were advertising was concealed before the study. Moreover, to make the participants believe that this study assessed the shopping experience and to ensure an appropriate interval between the interactions with the agents and the subsequent recall test, participants were instructed to explore the store thoroughly. They were encouraged to examine all the shelves attentively and spend at least 10 min from entering to leaving the store.

The study was conducted individually. The experimenter escorted each participant to the store entrance. Upon arrival, the participants collected a shopping basket. They walked slowly through the store and initially encountered Agent 1, who presented promotional content on fruit jelly. After listening to Agent 1, they continued their shopping expedition and subsequently listened to the promotion by Agent 2 on freeze-dried products. Upon concluding their shopping, participants proceeded to the cash register when they had items in their baskets. Those without any purchases returned their baskets and exited the store. Subsequently, they went to a predetermined meeting place and completed a free recall test using a Google Form. This test lasted 10 min, during which the participants were asked to write down as much as they could remember about what the agents had advertised and encouraged to continue answering even if they felt they could no longer recall details. After the test, the participants provided impression ratings for Agents 1 and 2, along with a general questionnaire regarding their overall experience.

## 4 Results

After reviewing the video recordings, we observed that six participants (one in the physical-same condition, two in the physical-different condition, one in the virtual-same condition, and two in the virtual-different condition) did not stop by the agents; consequently, their data were removed and not used in the analyses.

### 4.1 Recall

Initially, a correlation analysis was conducted to determine whether the age of participants affected recall performance; however, no significant correlation was identified, and the correlation coefficient was nearly zero (*t* (48) = −0.48 , *p* = 0.636, *r* = −0.07 ). Similarly, the recall performance did not show a significant correlation with the length of the interval between the first and second promotions (*t* (48) = 0.59, *p* = 0.559, *r* = 0.08) or with the length of the interval between the second promotion and the test (*t* (48) = 1.10, *p* = 0.279, *r* = 0.16). Thus, we performed the subsequently described analyses without considering these indicators.

**FIGURE 3:**
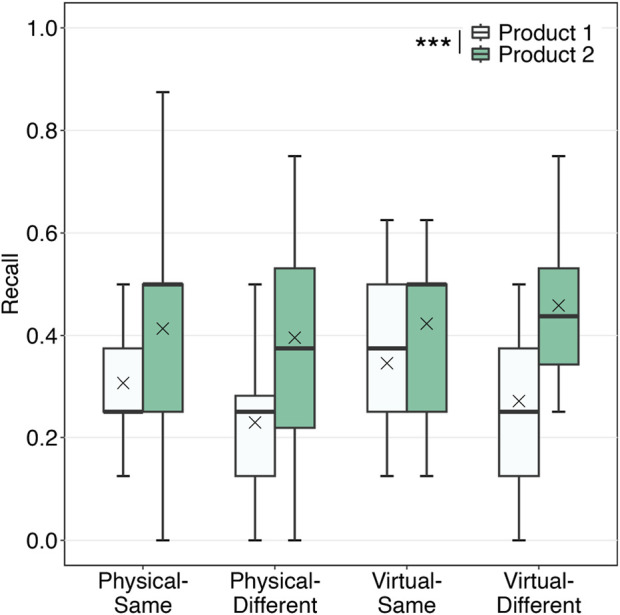
Proportion of correctly recalled information. The results of analyses are marked with asterisks (^***^: *p* < 0.001).

Figure 3 shows the proportion of correctly recalled information in the test. As the normality assumption was violated (Shapiro-Wilk test, *p* < 0.01), we first performed an aligned rank transformation (ART) and then conducted a modality × context × product mixed three-way analysis of variance (ANOVA). ART is a method that enables us to conduct an ANOVA for non-parametric data (Wobbrock et al., 2011). The addition of the product factor to the analysis, which was a within-participant variable, followed the prior study by Mizuho et al. (2023a). Splitting the observation has the advantage of providing more indications, such as examining the interaction between the multiple-context effect and the serial position effect [e.g., the recency effect (Isarida and Isarida, 2006)]. The results showed a significant main effect of the product factor (*F* (1, 46) = 16.71, *p* < 0.001, ${\eta_{p}}^{2}$ = 0.27): recall of the second product was superior to that of the first product. However, the results did not show a significant main effect of the modality (*F* (1, 46) = 0.44, *p* = 0.512, ${\eta_{p}}^{2}$ = 0.01), main effect of the context (*F* (1, 46) = 0.84, *p* = 0.365, ${\eta_{p}}^{2}$ = 0.02), two-way interaction effect of the modality and context (*F* (1, 46) = 0.14, *p* = 0.715, ${\eta_{p}}^{2}$ = 0.00), two-way interaction effect of the modality and product (*F* (1, 46) = 0.19, *p* = 0.665, ${\eta_{p}}^{2}$ = 0.00), two-way interaction effect of the context and product (*F* (1, 46) = 1.20, *p* = 0.280, ${\eta_{p}}^{2}$ = 0.03), or three-way interaction effect of the three factors (*F* (1, 46) = 0.02, *p* = 0.901, ${\eta_{p}}^{2}$ = 0.00).

### 4.2 Questionnaires on agents

Because three participants did not follow the instructions correctly when responding to the questionnaires, we excluded their data from the following analyses. For each indicator, if normality and homogeneity of variance could be assumed, we performed a mixed three-way ANOVA of modality (physical or virtual) × context (same or different) × agent (first or second). When the normality assumption was violated, we applied ART and conducted a three-way ANOVA. Only the key results are presented here to avoid complexity. Moreover, for each indicator, a correlation analysis was performed: First, for each participant, we computed the average of the subjective ratings for the two agents as a representative value. Similarly, we computed the mean of recall for the two products, and conducted a Pearson’s product-moment correlation test for each of the physical and virtual conditions, whose *p*-values were adjusted with the Holm method not to increase Type-I error.

#### 4.2.1 Social presence

**FIGURE 4:**
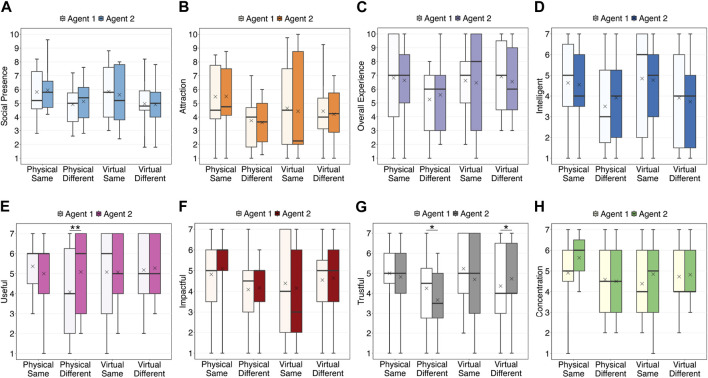
Ratings regarding the agents. The results of statistical tests are marked with asterisks (^**^: *p* < 0.01, ^*^: *p* < 0.05). **(A)** Social Presence. **(B)** Attraction. **(C)** Overall Experience. **(D)** Intelligence. **(E)** Usefulness. **(F)** Impactfulness. **(G)** Trustworthiness. **(H)** Concentration.

**FIGURE 5:**
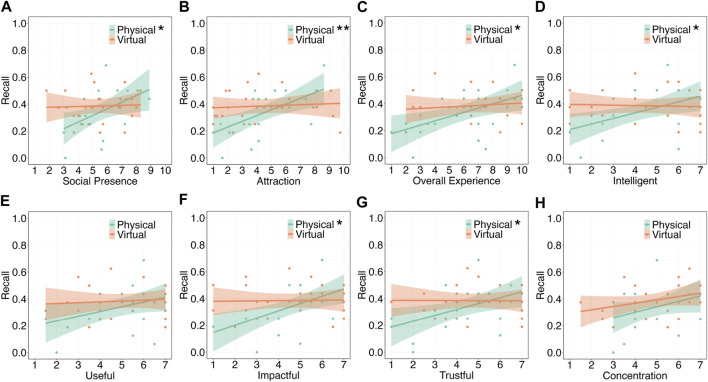
Correlation between recall and subjective ratings on the agents. The results of statistical tests are marked with asterisks (^**^: *p* < 0.01, ^*^: *p* < 0.05). **(A)** Social Presence. **(B)** Attraction. **(C)** Overall Experience. **(D)** Intelligence. **(E)** Usefulness. **(F)** Impactfulness. **(G)** Trustworthiness. **(H)** Concentration.

Figure 4A shows the degree of social presence. A three-way ANOVA did not show any significant differences between the conditions. Moreover, Figure 5A shows the regression lines between social presence and recall in the two modality conditions. The results showed a significant positive correlation under the physical condition (*t* (21) = 2.47, $p_{\mathrm{adj}}$ = 0.044, *r* = 0.47); however, no significant correlation was observed under the virtual condition (*t* (22) = 0.21, $p_{\mathrm{adj}}$ = 0.838, *r* = 0.04).

#### 4.2.2 Attraction

Figure 4B shows the attraction scores. A three-way ART-ANOVA did not show any significant differences between the conditions. Moreover, Figure 5B shows the regression lines between attraction and recall in the two modality conditions. The results showed a significant positive correlation under the physical condition (*t* (21) = 3.39, $p_{\mathrm{adj}}$ = 0.006, *r* = 0.59); however, no significant correlation was observed under the virtual condition (*t* (22) = 0.41, $p_{\mathrm{adj}}$ = 0.686, *r* = 0.09).

#### 4.2.3 Overall experience

Figure 4C shows the overall experience ratings. A three-way ART-ANOVA did not show any significant differences between the conditions. Moreover, Figure 5C shows the regression lines between the overall experience rating and recall in the two modality conditions. The results showed a significant positive correlation under the physical condition (*t* (21) = 2.74, $p_{\mathrm{adj}}$ = 0.024, *r* = 0.51); however, no significant correlation was observed under the virtual condition (*t* (22) = 0.60, $p_{\mathrm{adj}}$ = 0.553, *r* = 0.13).

#### 4.2.4 Intelligence

Figure 4D shows the intelligence scores. A three-way ART-ANOVA did not show any significant differences between the conditions. Moreover, Figure 5D shows the regression lines between intelligence and recall in the two modality conditions. The results showed a significant positive correlation under the physical condition (*t* (21) = 2.67, $p_{\mathrm{adj}}$ = 0.028, *r* = 0.50); however no significant correlation was observed under the virtual condition (*t* (22) = −0.23 , $p_{\mathrm{adj}}$ = 0.818, *r* = −0.05 ).

#### 4.2.5 Usefulness

Figure 4E shows the usefulness scores. A three-way ART-ANOVA showed a significant three-way interaction effect of the three factors (*F* (1, 43) = 11.07, *p* = 0.002, ${\eta_{p}}^{2}$ = 0.20). Subsequently, simple interaction effect and simple-simple main effect tests revealed that the scores for Agent 2 were significantly higher than that for Agent 1 under the physical different condition (*F* (1, 11) = 10.12, *p* = 0.009, ${\eta_{p}}^{2}$ = 0.48). No other significant effects were observed. Moreover, Figure 5E shows the regression lines between usefulness and recall in the two modality conditions. The results showed no significant correlation under the physical condition (*t* (21) = 1.93, $p_{\mathrm{adj}}$ = 0.133, *r* = 0.39) or under the virtual condition (*t* (22) = 0.42, $p_{\mathrm{adj}}$ = 0.678, *r* = 0.09).

#### 4.2.6 Impactfulness

Figure 4F shows the impactfulness scores. A three-way ART-ANOVA did not show any significant differences between the conditions. Moreover, Figure 5F shows the regression lines between impactfulness and recall in the two modality conditions. The results showed a significant positive correlation under the physical condition (*t* (21) = 3.09, $p_{\mathrm{adj}}$ = 0.011, *r* = 0.56); however, no significant correlation was observed under the virtual condition (*t* (22) = 0.12, $p_{\mathrm{adj}}$ = 0.904, *r* = 0.03).

#### 4.2.7 Trustworthiness

Figure 4G shows the trustworthiness scores. A three-way ART-ANOVA results showed a significant three-way interaction effect of the three factors (*F* (1, 43) = 13.43, *p* < 0.001, ${\eta_{p}}^{2}$ = 0.24). Subsequently, simple interaction and simple-simple main effect tests revealed that the scores for Agent 2 were significantly lower than those for Agent 1 under the physical different condition (*F* (1, 11) = 5.50, *p* = 0.039, ${\eta_{p}}^{2}$ = 0.33). Conversely, the scores for Agent 2 were significantly higher than those for Agent 1 under the virtual different condition (*F* (1, 10) = 5.46, *p* = 0.042, ${\eta_{p}}^{2}$ = 0.35). No other significant effects were observed. Moreover, Figure 5G shows the regression lines between trustworthiness and recall in the two modality conditions. The results showed a significant positive correlation under the physical condition (*t* (21) = 2.43, $p_{\mathrm{adj}}$ = 0.048, *r* = 0.47); however, no significant correlation was observed under the virtual condition (*t* (22) = −0.03 , $p_{\mathrm{adj}}$ = 0.973, *r* = −0.01 ).

#### 4.2.8 Degree of concentration

Figure 4H shows the degree of concentration while listening to the agents. A three-way ART-ANOVA showed that the main effect of the agent was marginally significant (*F* (1, 43) = 3.47, *p* = 0.069, ${\eta_{p}}^{2}$ = 0.07); however, no other differences were exhibited between the conditions. Moreover, Figure 5H shows the regression lines between the degree of concentration and recall in the two modality conditions. The results showed no significant correlation under the physical (*t* (21) = 1.70, $p_{\mathrm{adj}}$ = 0.104, *r* = 0.35) or under the virtual conditions (*t* (22) = 1.51, $p_{\mathrm{adj}}$ = 0.146, *r* = 0.31).

## 4.3 Questionnaires on shopping

### 4.3.1 Similarity to usual shopping experience

The similarity to the usual shopping experience was *M* = 4.31, *SD* = 1.97 under the physical same condition, *M* = 5.08, *SD* = 1.88 under the physical different condition, *M* = 4.61, *SD* = 1.89 under the virtual same condition, and *M* = 4.08, *SD* = 2.07 under the virtual different condition. After applying ART, the modality × context two-way ANOVA did not show the main effect of modality (*F* (1, 46) = 0.28, *p* = 0.596, ${\eta_{p}}^{2}$ = 0.01), main effect of context (*F* (1, 46) = 0.01, *p* = 0.910, ${\eta_{p}}^{2}$ = 0.00), or interaction effect of the two (*F* (1, 46) = 1.36, *p* = 0.250, ${\eta_{p}}^{2}$ = 0.03).

## 5 Discussions

### 5.1 Effects of multiple-agent promotion on customer memory

The main effect of modality, main effect of context, and their interaction effect were not significant with small effect sizes. Therefore, hypotheses H1, H2, and H3 were not supported. Furthermore, the means trended in the opposite direction of our hypotheses.

A possible explanation of the null results is that the store environment was highly stimulating, potentially causing the agents to blend into the overall sensory experience. This inference is supported by the relatively low levels of social presence, averaging around six out of ten. This notion also gains further credence from the fact that certain participants, who were later excluded from the analyses, had difficulty locating the agents and walked past them. Notably, these participants were explicitly provided with the locations of the agents on the map and instructed to listen to the advertisements. However, the stimulating store environment might have interfered with their ability to notice the agents. The store was filled with background music, other customers, and colorful merchandise. In contrast to our field study environment, previous studies that examined multiple-context effects and the distinctions between physical and virtual agents were predominantly conducted in controlled laboratory settings, which tend to be less stimulating and more conducive to the effectiveness of agents. Given these considerations, the impacts of agents should be determined in less stimulating commercial environments, such as the hallways in shopping malls (Song et al., 2021; Iwamoto et al., 2021), in future research.

The only significant effect observed in the recall was the main effect of the product, and this effect exhibited a large effect size. Several possible explanations were considered. First, product 2 was probably easier to remember than product 1 because we could not achieve a counterbalance of products by swapping the first and second products. This limitation was unavoidable because of the specific product placement arrangements within the store. Alternatively, participants might have recalled product 2 better because they might have mentally prepared themselves for the promotional details of the agent. This possibility aligns with the findings of Song et al. (2023), who observed that introducing an in-store robot via an out-of-store robot heightened the readiness of participants to engage with the in-store robot. Consequently, this increased preparedness may have improved the effectiveness of the recommendation by the in-store robot. Given the limited extent of validation regarding interactions with multiple agents in commercial settings, these considerations could offer valuable insights for future research.

### 5.2 Effects of agent modality on social attitudes in the field

No significant differences were observed in the social presence participants perceived toward the agents under the physical and virtual agent conditions. Thus, hypothesis H4 was not supported. Similarly, no significant differences were identified between the conditions for most subjective measures. Notably, previous laboratory experiments have consistently confirmed differences in social attitude based on agent modality (Li, 2015). Therefore, certain external factors may have interfered with the anticipated effects. As discussed in the previous section, a critical factor may be the overwhelming influence of the environmental stimuli experienced in the store.

Conversely, the fact that no clear difference was observed between physical and virtual agents in real-world settings holds practical value. If virtual agents, which are cost-effective, can achieve a comparable impact to physical robots, they should be a viable choice. Although this study was an initial step in deploying virtual agents in a commercial field, further studies will continue to compare the effectiveness of physical and virtual agents. Given that most real-world settings are more stimulating than controlled laboratory environments, robustly evident effects in a laboratory may probably manifest in a limited field, as observed in this study.

The significant differences observed in the subjective measures were exclusively present within the different context conditions. In the physcial-different condition, Agent 2 was perceived more useful but less trustworthy than Agent 1. Conversely, in the virtual-different condition, Agent 2 was perceived more trustworthy than Agent 1. While we can not explain the results because of lacking previous studies employing multiple agents, this outcome suggests that the variation in the deployed agents allowed us to change the environmental context of the participants to some extent. Conversely, it is interesting that even when participants encountered the same agent twice, their responses to the second agent did not vary dramatically. However, our measurement approach had a limitation, as participants collectively answered the questionnaire after leaving the store. Thus, our results might have differed if we had administered the questionnaire immediately after each interaction with the agents. Because research involving multiple agents is still relatively new, these findings will offer valuable insights for guiding future research endeavors.

### 5.3 Significant correlation between social attitudes toward physical agent and recall

Under the physical agent condition, we identified a significant positive correlation between social presence and recall. However, no such trend was observed in the virtual agent condition. Thus, hypothesis H5 was partially supported. This pattern of results was mirrored in the assessments related to attraction, overall experience, intelligence, impactfulness, and trustworthiness. These consistencies can be explained by the findings of Lee et al. (2006a); Lee et al. (2006b), who indicated that social presence mediates other subjective feelings toward agents.

Notably, the physical and virtual agent conditions exhibited different trends in the relationship between social attitudes and recall. Although subjective indicators measured by questionnaires, including social presence, did not show significant differences between agent modalities, whether agent is physically present or not may affect participants’ cognition as suggested by previous studies (Li, 2015) and may result in some differences in memory processes. Further investigation is needed to identify mechanisms, including observing behavioral indicators such as eye gaze. According to the positive correlation under the physical agent condition, improving the social presence of agents can potentially enhance recall performance. To increase the social presence, conducting a verification in a less stimulating environment would be one solution, as discussed in the previous sections. Conversely, when looking at the scatter-plot in Figure 5, participants who rated social presence with lower scores tended to exhibit lower recall performance. Thus, the use of a robot agent that lacks well-designed gestures (Kose-Bagci et al., 2009) may result in diminished social presence and, in turn, reduced recall. Conversely, when virtual agents are employed, social attitudes toward the agent may be unrelated to recall, which suggests that utilizing virtual agents may be a viable option if resources cannot be allocated for fine-tuning agent design. However, the current analyses are based on correlation and do not reveal causal relationships. Future research endeavors that manipulate social presence as an independent variable, for instance, by modifying the designs of gaze and gesture, may provide valuable insights and further elucidate the relationship between social attitudes and recall.

## 6 Limitations and future work

A critical limitation of this study is that it did not involve general customers but relied on experimental collaborators who were recruited. To preserve the realism of the shopping experience, we did not disclose our use of agents for advertising or our intent to assess memory at a later stage. Consequently, participants rated their experience with an average of four out of seven in terms of similarity to their typical shopping experience. Additionally, half of the participants actually purchased something in the field study, while a few bought the products promoted by the agents: one bought product 1, and four bought product 2. Notably, some special instructions were inevitable to assess memory appropriately. The most prominent instruction was ensuring that participants listened to the advertisements presented at two locations. While the agents were positioned in easily visible areas, the passage of customers by these specific locations could not be guaranteed. Moreover, whether customers would stop and actively engage with it was uncertain. Therefore, without this instruction, there was a risk of a reduced number of valid participants who had heard both advertisements. Simultaneously, this issue underscores a practical weakness concerning the feasibility of the proposed method. Nevertheless, this problem can be mitigated by strategically placing agents in areas where customers are bound to pass by, such as at the entrance and just before the register. However, when conducting this field study, such placements were not possible based on the specifications of the store owner. The challenge of customers not stopping at agents can be partially resolved by optimizing agent behavior (Okafuji et al., 2022), and this issue may be addressed by placing agents in areas where customers are more likely to come to a standstill, such as elevator halls. We plan to test the generalizability and feasibility of the findings by testing in various locations.

Another notable limitation is that we exclusively employed the humanoid robot Sota as the agent. Given that Sota is a relatively small robot, a larger robot might have had a more pronounced presence in the noisy environment of a grocery store. Conversely, large agents can be obtrusive and reduce the available space for product placement, which is a disadvantage. Additionally, although physical co-presence generally has positive effects, as supported by the review (Li, 2015), it is important to note that in certain situations, it may also have negative effects (Kose-Bagci et al., 2009; Shinozawa et al., 2005). Moreover, virtual agents are better suited than physical robots in terms of the variety of agent types. In this study, Sota was shown on the display to examine the modality effect, but the appearance of the virtual agents can be easily customized. In a previous study on the multiple-context effect, Mizuho et al. (2023a) used various virtual agents, including a red panda, an extraterrestrial being, a robot, and a human. The current study did not exhibit any significant disadvantages associated with the experience or cognition of using virtual agents when compared to physical agents. Therefore, investigating the use of virtual agents, particularly considering the impact of agent appearance, holds promise for future research endeavors.

Additionally, there were other methodological limitations. One key issue is the sample size and statistical power. As described in Section 3.4, we pre-determined the sample size using G*Power. However, the observed effect size was smaller than expected, which may have resulted in insufficient detection power. Based on the effect sizes found in this study, future research could design studies with a more appropriate sample size. Moreover, participants were not informed in advance of the agent’s presence, which may have impacted the results due to an element of surprise. This approach was intended to replicate a natural shopping experience, but different outcomes may emerge under conditions where participants are highly familiar with agents, a scenario that could become relevant as agent-based advertising becomes more widespread. Another limitation is that the evaluation of participants’ social attitudes toward the agent relied entirely on subjective questionnaire responses. While incorporating objective indicators, such as gaze tracking, would require additional setup and incur higher implementation costs, it would allow for a more comprehensive assessment. Moreover, measuring attention to the agent in this way may offer insights into whether the observed effects on memory result from ease of recall, as suggested by the multiple-context effect, or rather from ease of encoding when encountering the agent. Furthermore, qualitative methods, such as thematic analysis of verbal interview data, could enhance our understanding by providing multi-dimensional insights into the participants’ experiences.

Lastly, from the implications of this study, we summarize the hypotheses for future field research examining the impact of agent-based promotion on customer memory. (1) It is essential to design the experiment carefully considering how much social presence of agents is ensured in a field. The differences in social presence between the physical and virtual agents that had been observed in the laboratory experiments were not observed in the present field study. Therefore, it is necessary to deliberate on the influence of the situation surrounding the agent, which is often overlooked in laboratory experiments. In addition, a positive correlation between social presence and memory performance was observed in the robot condition in the present study. Thus, we may be able to enhance recall by increasing the social presence of robots in the field, such as adjusting the placement of the robot or exaggerating its behavior. (2) Focusing on the potential of virtual agents is also a promising avenue. This was the first study where virtual agents were placed in a real store environment (not an online store) and product promotions were implemented. We did not observe any negative effects of virtual agents on social attitudes or memory retention. Therefore, we expect to utilize virtual agents further, which are more cost-effective than robots. We can also consider approaches that are unique to virtual agents, such as employing agents with various appearances. (3) It would be worthwhile to examine the effect of advertising by multiple agents. There is still a possibility that the multiple context effect that this study aimed for can be obtained in situations with high social presence or by expanding the difference between the two agents. In this study, the difference between the two types of agents was represented by changing the appearance of the same type of robot; however, this is unlikely to be sufficient to produce the multiple-context effect. The result that the second agent’s advertisement was better remembered may also be related to the “readiness to listen to the advertisement from the robot” in the Song et al. study (Song et al., 2023). Although managing multiple agents is labor intensive, we believe it is worth investigating.

## 7 Conclusion

In this study, we placed two agents in a grocery store to evaluate how well customers remembered what the agents advertised. We manipulated the variability of the agents, that is, if they were identical or distinct. We also compared the effects of placing physical robots or virtual agents. The results did not show the effects of modality or variability of agents. However, when observing the relationship between the social presence of agents and memory performance, we found a significant positive correlation when using the physical robots: the higher the social presence, the higher the memory performance. Conversely, no such trend was observed for virtual agents. To the best of our knowledge, this study was the first study in which virtual agents were placed in the field to advertise. It also assessed customer memory, an important but largely untested area for advertising. This study would serve as a first stepping stone in these new endeavors.

## Data Availability

The raw data supporting the conclusions of this article will be made available by the authors, without undue reservation.

## Footnotes

1
https://www.vstone.co.jp/products/sota/

2
https://voicevox.hiroshiba.jp/
